# The Function of NF-Kappa B During Epilepsy, a Potential Therapeutic Target

**DOI:** 10.3389/fnins.2022.851394

**Published:** 2022-03-10

**Authors:** Mengtan Cai, Weihong Lin

**Affiliations:** Department Neurology, Bethune First Hospital of Jilin University, Jilin University, Changchun, China

**Keywords:** epilepsy, NF-kappa B, inflammation, chemokines, interleukins, tumor necrosis factor

## Abstract

The transcriptional regulator nuclear factor kappa B (NF-κB) modulates cellular biological activity by binding to promoter regions in the nucleus and transcribing various protein-coding genes. The NF-κB pathway plays a major role in the expressing genes related to inflammation, including chemokines, interleukins, and tumor necrosis factor. It also transcribes genes that can promote neuronal survival or apoptosis. Epilepsy is one of the most common brain disorders and it not only causes death worldwide but also affects the day-to-day life of affected individuals. While epilepsy has diverse treatment options, there remain patients who are not sensitive to the existing treatment methods. Recent studies have implicated the critical role of NF-κB in epilepsy. It is upregulated in neurons, glial cells, and endothelial cells, due to neuronal loss, glial cell proliferation, blood-brain barrier dysfunction, and hippocampal sclerosis through the glutamate and γ-aminobutyric acid imbalance, ion concentration changes, and other mechanisms. In this review, we summarize the functional changes caused by the upregulation of NF-κB in the central nervous system during different periods after seizures. This review is the first to deconvolute the complicated functions of NF-κB, and speculate that the regulation of NF-κB can be a safe and effective treatment strategy for epilepsy.

## Introduction

Epilepsy is among the most common brain disorders (Scheffer et al., 2017) and is characterized by repeated convulsions due to abnormal excitation of neurons (Wijnen et al., 2017). It is estimated that 50–70 million people (Trinka et al., 2019), accounting for approximately 1–2% of the world’s population, are affected by epilepsy. According to a survey, approximately 70% of people with epilepsy can have normal lives if they are adequately treated with anti-epileptic drugs (World Health Organization, 2005). However, anti-epileptic drugs used by patients block epileptic seizures but do not affect the underlying pathology or disease progression. Recently, some studies have highlighted the important pathophysiological role of inflammation in epilepsy (Paudel et al., 2019) and revealed that excessive activation of inflammatory pathways is a sign of epilepsy (Marcheselli and Bazan, 1996; Russmann et al., 2017). Studies have demonstrated that brain injury-induced inflammation and apoptosis are causative factors of epilepsy (Kwon et al., 2013); therefore, seizures can also cause the expression of inflammatory factors. These mediators can trigger the activation of the nuclear factor kappa B (NF-κB) pathway, and the activated NF-κB can, in return, promote their transcription.

Nuclear factor kappa B encodes various proteins that play a crucial role in immunity, inflammation, cell growth, survival, and apoptosis (Singh and Singh, 2020). Moreover, NF-κB up-regulation can increase the expression of pro-inflammatory cytokines during the proliferation of hippocampal glial cells (Wang et al., 2017), and this plays a crucial role in epilepsy. The NF-κB transcription factor family in mammals consists of five proteins (Oeckinghaus and Ghosh, 2009) and can be subdivided into the Rel subfamily [including c-Rel, Rel B, and Rel A (aka p65)] and the NF-κB subfamily (including p105/p50, p100/p52 and so on), both of which have a Rel homology domain (Mitchell et al., 2016). Rel proteins have a C-terminal transactivation domain that can activate transcription. Some proteins in the NF-κB subfamily that cannot function as transcriptional activators become shorter (p105 to p50 and p100 to p52) through limited proteolysis (Gilmore, 2006). Normally, NF-κB exists in an inactive state as a dimer (usually p50 and p65) bound to an inhibitor (IκB) (Karin, 1999) in the cytoplasm. Theoretically, there are 15 possible combinations of dimers but only 13 dimers are known to exist in cells (Zhang et al., 2017). The IκB protein family consists of IκBα, IκBβ, IκBγ, IκBδ, IκBε, and Drosophila Cactus, among others. When the cell receives a stimulus, there is an intracellular activation of the IκB kinase (IKK) complex comprising catalytic kinase enzymes including IKK-α, IKK-β, and IKK-γ/NF-κB essential modulator, which further integrates the downstream activating signals by phosphorylating the inhibitor of NF-κB (Mitchell et al., 2016).

Two types of NF-κB signal transduction pathways have been identified. In the classical pathway, NF-κB is activated by extracellular stimuli such as Tumor Necrosis Factor-α (TNF-α), which activates cell surface receptors and recruit adaptor proteins through the cytoplasmic domain, triggers a signaling cascade that ultimately activates the IKK complex through phosphorylation of the serine of IKKβ (Ser177 and Ser181). The activated IKK complex phosphorylates IκBα (at Ser32/Ser36 on IκBα) (Kendellen et al., 2014), which is degraded by ubiquitination. This causes NF-κB dimers (mostly p65 and p50), which actively shuttle between the nucleus and cytosol, to stay nuclear and induce gene expression. In contrast with this, the signal-related adaptor protein, in the non-canonical pathway, can only be recruited by some molecules, such as CD40, and ubiquitination is not required. In this pathway, IKKα is phosphorylated, and p100 is recruited leading to the phosphorylation of p100 by IKKα, partial hydrolysis, and conversion to p52 to expose its nuclear localization signal and DNA binding domain. It can form a complex with Rel B in the human nucleus to activate the target gene expression (Verma et al., 2019). Cross-talk exists between activation pathways. Both Rel B and p100 genes contain κB binding sites, and their transcription is dependent on p65 (Bren et al., 2001). p100, also called IκBδ, can inhibit p65 (Shih et al., 2011). When p52 is lacking, Rel B forms a dimer with p50. Similarly, when p50 is lacking, p65 forms a dimer with p52, which has almost the same level of p65 activation and target inflammatory gene expression (Hoffmann et al., 2003). IκBα is a target gene of NF-κB, and re-expressed IκBα inhibits NF-κB activity. Other target genes, such as interleukins (ILs) and TNF-α, can block IKK-dependent phosphorylation of IκBα to inhibit NF-κB activation. Studies have found that the NF-κB pathway can stay active for 30–60 min in most cells (Qin et al., 2007) and be activated under several conditions that can promote the transcription of target genes that affect the function of neurons.

Nuclear factor kappa B overexpresses various genes implicated in oxidative stress and inflammatory diseases and can regulate neurogenesis, neuronal death survival, and synaptic plasticity (Buckmaster and Dudek, 1997; O’Neill and Kaltschmidt, 1997). Brain tissue analysis of epileptic patients and animal models has shown that NF-κB has complex functions related to neuron survival and injury (Mattson, 2005). Therefore, targeting of NF-κB by selective or non-selective inhibitors or agonists in epilepsy can serve as an attractive therapeutic approach.

## Changes of Nuclear Factor Kappa B Expression in Status Epilepticus

Increased NF-κB expression has been observed in brain tissue from both animal models (Lerner-Natoli et al., 2000) and patients with epilepsy (Lubin et al., 2007; Table 1).

**TABLE 1:** NF-κB proteins with abnormal expression and/or activity associated with epilepsy.

Author	Year	Country	Treatment drug	Time after treatment	Method	Type of NF-κB	Species	Brain areas
Qu et al. (Qu et al., 2019)	2019	China	Lithium-pilocarpine	24h	qRT-PCR, WB	p65	SD rats	Hippocampus
Shi et al. (Pérez-Otano et al., 1996)	2018	China	–	–	IHC	p65	Human	Brain
Ojo et al. (Ojo et al., 2019)	2019	Nigeria	Kainic acid	6h	IHC	p65	Swiss rats	Hippocampus
Singh et al. (Bai et al., 2018)	2018	India	Pentylenetetrazole	–	WB	–	Wistar rats	Hippocampus
Mohamed et al. (Singh et al., 2018)	2020	Egypt	Pentylenetetrazole	14d	ELISA	–	Wistar rats	Hippocampus
Wang et al. (Wang et al., 2017)	2017	China	Lithium-pilocarpine	1,7,14,30,60d	qRT-PCR	–	SD rats	Hippocampus
Blondeau et al. (Rosciszewski et al., 2019)	2001	France	Kainic acid	1,24,72h	WB	p50, p65	Wistar rats	Hippocampus
Ryu et al. (Lanzillotta et al., 2010)	2011	Korea	Lithium-pilocarpine	3-4d	IHC	p65-Ser536 (while p52-Ser865, p52-Ser869, p65-Ser276, p65-Ser311, p65-468,p65-Ser529 were decreased in degenerating neurons)	SD rats	Hippocampus
Won et al. (Kim and Kang, 2017)	1999	Korea	Kainic acid	0.5,4,8,24,72h	IHC,WB	p50	SD rats	Hippocampus
Firdous et al. (Soerensen et al., 2009)	2021	Pakistan	Pentylenetetrazole	–	ELISA	pNF-κB	SD rats	Cortex, Hippocampus
Miller et al. (Carrasco et al., 2000)	2014	United States	Kainic acid	24h	IF	–	cis- NF-κB^EGFP^ transgenic reporter mice	Hippocampus
Paudel et al. (Paudel et al., 2020)	2020	Malaysia	Pilocarpine	10d	RT-PCR	–	Zebrafish	Brain

*d, days; ELISA, enzyme linked immunosorbent assay; h, hours; IF, immunofluorescence; IHC, immunocytochemistry; qRT-PCR; Real-Time Quantitative Reverse Transcription Polymerase Chain Reaction; RT-PCR, Reverse Transcription-Polymerase Chain Reaction; Ser, Serine; SD rats, Sprague Dawley rats; WB, Western-Blot.*

In animal models, the increase in NF-κB first occurs in neurons after seizures. The first report demonstrating the expression of NF-κB in the rat brain showed that the activity of NF-κB (p50) in hippocampal neurons increases rapidly (within 4 h) in response to epilepsy induced by pentylenetetrazole (Prasad et al., 1994). Another study also showed a significant upregulation of NF-κB 4 h after kainic acid (KA) injection that peaked 8–16 h post-treatment (Rong and Baudry, 1996). Given the different cells in the brain, Lerner-Natoli et al. (2000) found that the changes in NF-κB expression first occurred in the hippocampal neurons 24 h after KA injection, which was earlier than that in glial cells. Thus, the NF-κB upregulation at this timepoint in neurons was independent of glial cells and may be related to calcium influx (Furukawa and Mattson, 1998) caused by the activation of N-methyl-D-aspartic acid (NMDA) and α-amino-3-hydroxy-5-methyl-4-isoxazole-propionic acid receptors.

Nuclear factor kappa B expression increased in glial cells later than its change in neurons. Pérez-Otano et al. (1996) proposed that NF-κB expression increased in astrocytes 2 days after KA injection. Matsuoka et al. (1999) found that NF-κB expression was upregulated in blood vessels and glial cells, but it disappeared in vertebral neurons after 1 day of status epilepticus (SE) induced by KA microinjection Similarly, Lerner-Natoli et al. (2000) demonstrated that overexpression and increased activation of NF-κB occurred in thickened astrocytes 4–8 days after SE. Previous studies have proposed that the activation of inflammatory signals in glial cells is involved in KA-induced neurodegeneration, which suggests that the activated NF-κB in glial cells participates in the delayed and long-term response of glia to injury.

Astrocytes and microglia have been extensively studied in epilepsy. However, the cell types that overexpress NF-κB have not been established. Pérez-Otano et al. (1996) and Lerner-Natoli et al. (2000) both found that the number of microglia increased after SE but NF-κB was expressed in astrocytes. On the other hand, Kim and his group found an increase in phosphorylated NF-κB at the threonine 435 site in microglia after SE (Kim et al., 2019). Similarly, NF-κB serine 276 phosphorylation was found to be increased in microglia in the frontoparietal cortex (Kim et al., 2020) or piriform cortex (Lee et al., 2014). The elevated high mobility group protein B1 after epilepsy must activate microglia through the toll-like receptor 4 (TLR4)/receptor for advanced glycation endproducts for late glycation end products of the NF-κB pathway to disrupt the function of neurons (Shi et al., 2018; Massey et al., 2019; Rosciszewski et al., 2019). Currently, there is no literature on NF-κB expression in oligodendrocytes and NG2 cells (polydendrocytes) in epilepsy.

**FIGURE 1:**
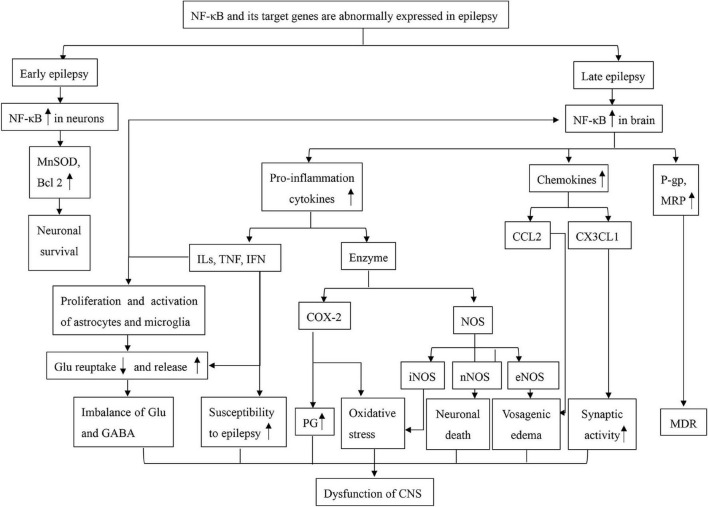
A mind map of NF-κB dysfunction in epilepsy. Bcl2, B-cell lymphoma-2; CCL2, chemokine (C-C motif) ligand 2; COX-2, cyclooxygenase 2; CX3CL1, fractalkine; Glu, glutamate; GABA, γ-aminobutyric acid; ILs, interleukins; IFN, interferons; MnSOD, manganese-superoxide dismutase; MRP, multidrug resistance protein; MDR, multiple drug resistance; NF-κB, nuclear factor kappa B; NOS, nitric oxide synthase; eNOS, endothelial nitric oxide synthase; iNOS, inducible nitric oxide synthase; nNOS, neuronal nitric oxide synthase; PG, prostaglandin; P-gp, P-glycoprotein; TNF, Tumor Necrosis Factor.

There has also been controversy about how long NF-κB persists after SE. Voutsinos-Porche et al. (2004) and his group found that the immunohistochemical expression of NF-κB began increasing 12 h post-injection and returned to basal levels by 3 and 6 days. In contrast, Wang et al. (2017) found that the expression of NF-κB in the epileptic hippocampus was highest on the 14th day after SE; on the 60th day the expression of NF-κB in the epilepsy group was higher than that in the controls Despite these inconsistencies, the close relationship between inflammation and epilepsy has been confirmed. Thus, as a major regulatory factor of inflammation, NF-κB plays an important role in the occurrence and development of epilepsy (Figure 1).

## Abnormal Expression of Nuclear Factor Kappa B and Its Target Genes Can Affect Neuronal Function and Survival

### Nuclear Factor Kappa Band Neuronal Survival

After establishing that NF-κB expression changes in epilepsy, several studies related to NF-κB function in neurons have emerged. When neuronal cells were exposed to external stimuli related to NF-κB, such as TNFα pretreatment (Furukawa and Mattson, 1998) or activation of TNF receptors (Marchetti et al., 2004), their survival rates could increase. Rel A knockout (Carrasco et al., 2000) or IκBα overexpression in cells induced apoptosis by NF-κB elimination (Mattson and Camandola, 2001), which suggests that NF-κB actively inhibits cell death signaling. At the same time, some studies have suggested that the upregulation of NF-κB plays a neuroprotective role in the short-term after seizures in animal models (Rong and Baudry, 1996; Mattson et al., 1997; Carrasco et al., 2000; Lerner-Natoli et al., 2000; Blondeau et al., 2001; Lanzillotta et al., 2010), which may be due to an increase in the expression of proteins associated with neuronal survival. Singh et al. concluded that NF-κB activation in neurons during epilepsy can promote the expression of B-cell lymphoma-2 (Bcl2) (Singh and Singh, 2020), and Mattson et al. showed that manganese-superoxide dismutase and Bcl2, which were necessary for neuronal plasticity and physical activity, were the target genes of NF-κB (Mattson and Camandola, 2001). Since NF-κB has different subunits, studies are now investigating whether the phosphorylation of different subunits has different effects. The phosphorylation mentioned in the above studies has almost always been of the p65 subunit. However, Ryu et al. (2011a) and Kim et al. (2013) showed that the phosphorylation of p52 can promote the expression of Bcl2, the phosphorylation of p65-Ser529 participates in cell growth, while phosphorylation at other sites on p65 may be related to cell death and inflammation. Moreover, c-Rel can induce apoptosis and is strongly activated in apoptotic neurons (Abbadie et al., 1993). We cannot know the exact NF-κB subunit upregulated in neurons during early epileptic onset, but there is no doubt that it is associated with neuron survival.

### Dysfunctions of Nuclear Factor Kappa B and Neurons

Nuclear factor kappa B is a major inducer of pro-inflammatory cytokines (Mattson, 2005), which can cause increased spontaneous epileptic recurrence and encode neurotoxic substances. NF-κB acts as a key point of convergence for multiple stress signals, including pro-inflammatory cytokines and oxidative stress (Miller et al., 2014). The target genes of NF-κB associated with epilepsy include cytokines, enzymes, and receptors, among others (Grilli and Memo, 1999).

#### Nuclear Factor Kappa B Inflammation, Abnormal Neuronal Function, and Apoptosis

The molecules induced by NF-κB are mainly divided into ILs, interferons (IFNs), TNF superfamily, colony-stimulating factors, and some enzymes, which can cause neuronal death by inducing inflammation. Among these, the most widely studied are ILs, TNFs, cyclooxygenase 2 (COX-2), and nitric oxide synthase.

Pro-inflammatory cytokines, such as IL-1α, IL-1β, IL-6, IL-10, TNF-α, and IFN-γ, are usually released by activated microglia and astrocytes (O’Neill and Kaltschmidt, 1997; Vezzani and Baram, 2007) and typically concentrated in low quantities within the brain. Their expression increased after seizures (Scorza et al., 2018), and they can enhance neuronal excitability and form a toxic microenvironment that promotes the progression of epilepsy (Kovács et al., 2011; Huang et al., 2021). The imbalance between glutamate and γ-aminobutyric acid (GABA) in the brain after epileptic seizures depends on the action of these cytokines (Hu et al., 2000; Takeuchi et al., 2006), and they may cause neuronal death through mitochondrial dysfunction or abnormal ion currents (Folbergrová and Kunz, 2012). Particularly, TNF-α has been found to increase microglial glutamate (Takeuchi et al., 2006) and induce GABA receptor endocytosis (Stellwagen et al., 2005). Moreover, IL-1β, TNF-α, and IFN-γ inhibit glutamate reuptake in astrocytes, and this can be blocked by IFN-β (Hu et al., 2000). The activation of TLR4/NF-κB in glial cells increases susceptibility to epilepsy (Iori et al., 2013) and is possibly associated with increased concentrations of IL-1β (Dubé et al., 2005), TNF-α (Balosso et al., 2005), IL-6 (Fukuda et al., 2007), and IL-2 (De Sarro et al., 1994). NF-κB target genes can also, in turn, activate it through corresponding receptors on neurons and glial cells to promote the expression of pro-inflammatory factors (Listwak et al., 2013), which further aggravates brain injury. These pro-inflammatory factors can also have beneficial effects, such as increased survival rates, observed in TNF-α-pretreated neurons in response to harmful stimuli; these effects may be mediated by NF-κB (Furukawa and Mattson, 1998).

The most widely studied enzymes induced by NF-κB in glial cells during epilepsy include COX-2 and nitric oxide synthase. After SE, the expression of COX-2 in glial cells and neurons increases, and it is often considered a marker of inflammation (Drion et al., 2018). COX-2, which is expressed at low to moderate levels in the cell bodies and dendritic spines of hippocampal neurons, is shown to be modulated by NF-κB (Yamagata et al., 1993) in neurons during SE (Yagami et al., 2016; Arena et al., 2019). COX-2 expression is regulated by synaptic activity (Kaufmann et al., 1996) and corresponds to the role of NF-κB in synaptic transmission (Yamagata et al., 1993). COX-2 inhibition prevents long-term potentiation of the perforant-path synapse to the dentate granule cells (Chen et al., 2002). After selective inhibition of COX-2, the excitability of hippocampal CA1 pyramidal neurons and dendritic membranes significantly reduced, which may be due to alterations in potassium currents (Chen and Bazan, 2005). COX-2 can also transform arachidonic acid into prostaglandin and other harmful substances to promote inflammation (Xu et al., 2020). At the same time, antiepileptic treatments can reduce the COX-2 in glial cells (Drion et al., 2018). Neuronal nitric oxide synthase, which is primarily expressed in neurons (Cinelli et al., 2020), can trigger pentylenetetrazole kindling epilepsy-induced endoplasmic reticulum stress and oxidative damage (Zhu et al., 2017). It can oxidize amino acids to produce nitric oxide (NO) and promote inflammation, like other nitric oxide synthases (Dreyer et al., 2004), as well as mediate glutamate-induced neuronal death and cause mitochondrial damage (Brown and Bal-Price, 2003). Activated microglial cells can kill neurons *via* NO from inducible nitric oxide synthase (iNOS) by inhibiting neuronal respiration, rapid glutamate release from both astrocytes and neurons, and subsequent excitotoxic death of the neurons (Brown and Bal-Price, 2003). Both COX-2 (Mazumder et al., 2017) and iNOS (Tawfik et al., 2018) cause oxidative stress, and they may be related to neuronal apoptosis induced by mitochondria (Wang et al., 2019).

#### Circulation Between Chemokines and Nuclear Factor Kappa B in Epilepsy

Chemokines are small cytokines or signaling proteins secreted by cells, with a molecular weight of approximately 8–10 kDa, and they can be regulated by NF-κB. They have four conserved cysteine residues to ensure their tertiary structure and can be divided into the following four major subfamilies: CXC, CC, CX3C, and XC (Fu et al., 2010; Huang et al., 2014, 2017; Karimian et al., 2017). Astrocytes, resident microglia, and endothelial cells have been identified as the cellular source of chemokines in the central nervous system under physiological and pathological conditions (Ambrosini and Aloisi, 2004; Fabene et al., 2010; Bozzi and Caleo, 2016). As their receptors are usually expressed on glial cells, chemokines affect epilepsy by regulating glial function. Several chemokine variants are known to alter neuronal physiology by modulating voltage-dependent channels, activating g-protein-gated potassium inflow channels, and increasing the release of certain neurotransmitters (Fabene et al., 2010). Wang et al. (2016) found that the levels of chemokine (C-C motif) ligand 2 (CCL2) and CC chemokine receptor 2 were increased in patients with intractable epilepsy. An increase in CC chemokine receptor 2 was also observed in CD68 + microglia, which can be caused by seizures (Bozzi and Caleo, 2016). Fractalkine (CX3CL1) is a transmembrane chemokine expressed by neurons (Pawelec et al., 2020) and glial cells (Ali et al., 2015). Microglia maintain the neurogenic niche environment through the interaction with neurons *via* fractalkine signaling under physiological conditions (Araki et al., 2021). However, under pathological conditions, the CX3CL1/chemokine (C-X3-C motif) receptor 1 axis can alter synaptic activity in epilepsy through microglia (Wake et al., 2009), and the upregulation of CX3CL1 can decrease GABA currents through the GABAA receptor (Roseti et al., 2013). Temporal lobe epilepsy patients also exhibit increased chemokine (C-X-C motif) ligand and (CXCL) 4 receptor expression in microglia and astrocytes, which eventually increase the glutamate levels (Lee et al., 2007). The increase in CXCL2 mRNA production has been demonstrated in epilepsy (Hu et al., 2020), and knockout of the CXCL2 receptor gene in astrocytes can affect the functioning of the blood-brain barrier (BBB) (Liu X. X. et al., 2020). Chemokines and their receptor genes are targeted by NF-κB (Fu et al., 2010; Duan et al., 2014; Ni et al., 2019), and chemokine receptors can also activate NF-κB (Ding et al., 2016; Bai et al., 2018; Zhuang et al., 2019), which may serve as a vicious circle in epilepsy.

#### Functional Crosstalk Between Nrf2 and Nuclear Factor Kappa B Inhibits the Expression of Antioxidant Molecules

Nuclear factor (erythroid-derived 2) -like 2 (Nrf2) is a transcription factor that can enhance the expressions of several antioxidants (Singh et al., 2018). In epilepsy models, Nrf2 expression reduction has been observed (Geng et al., 2018), but the ablation of the Nrf2 gene also enhances the degradation of IκBα, which leads to increased activation of the NF-κB protein and further upregulation of the expression of pro-inflammatory cytokines (Thimmulappa et al., 2006). Another mechanism is the competition between Nrf2 and NF-κB for the transcriptional coactivator creb binding protein complex (Singh et al., 2019). Injury-induced increase in NF-κB expression limits the availability of creb binding protein for Nrf2 complex formation and leads to an increase in the expression of NF-κB-driven inflammatory genes (Wardyn et al., 2015). Moreover, the decreased expression of antioxidant molecules may lead to the aggravation of neuronal damage caused by oxidative stress, which further affects the function of the nervous system.

#### Other Factors Influencing Neuronal Apoptosis Pathway Associated With Nuclear Factor Kappa B

As a key regulator of apoptosis, increased levels of NF-κB in the hippocampus inevitably lead to increased levels of caspase-3, which is associated with apoptosis (Mohamed et al., 2020). The excitotoxicity of neurons caused by overstimulation of NMDA receptors can directly activate NF-κB and cause cell apoptosis (Engelmann et al., 2014), which may be related to calcium influx (Vezzani et al., 2008).

## Abnormal Activation of Nuclear Factor Kappa B Can Affect Glial Cell Activity Through Proliferation and Polarization

The following are the four main types of glial cells: astrocytes, microglia, oligodendrocytes, and nerve/glial antigen 2 cells (polydendrocytes) (Pitkänen and Lukasiuk, 2009). Of these, astrocytes and microglia are the most widely studied in epilepsy. Overactivation of NF-κB in glial cells can cause changes in central nervous system function, promote seizures, and affect cellular activity, proliferation, and polarization of the glial cell.

The pro-inflammatory factors in epileptic models are mainly expressed through glial cells, which, in turn, affect the activity of neurons. Because of the presence of related receptors, the changes in the expressions of these molecules can affect the function of the glial cells. TNF-α stimulation of primary astrocytes *in vitro* can promote cell proliferation and viability and increase the expressions of NF-κB, P-glycoprotein (P-gp), and multidrug resistance-associated proteins 1 (Wang X. et al., 2018). The latter two are associated with drug resistance in epilepsy. TNF-α and IL-1β can promote the activity and proliferation of astrocytes (Cui et al., 2011) and microglia (Bruttger et al., 2015; Zhao et al., 2018) and inhibit glutamate reuptake into primary astrocytes (Ye and Sontheimer, 1996), thereby aggravating seizures. IL-1 receptor-associated kinase 1 is also regulated by NF-κB (Deng et al., 2019), which can also activate NF-κB to produce pro-inflammatory effects in glial cells (Liu G. J. et al., 2020). Chemokines such as CCL2 can also promote primary cultured microglia proliferation (Zhang et al., 2018).

Microglia activation plays a key role in regulating inflammation and immune response and can have a pro-inflammatory or anti-inflammatory effect depending on the M1/M2 polarization phenotype (Zhao et al., 2019). The phosphorylation of p65-Ser276 can convert microglia from the resting state to the activated state (Kim et al., 2020). The increase of p65 in microglia can promote the transition to the M1 phenotype of microglia (Zhang et al., 2019). Activated M1 microglia can release IL-1α and TNF, which can induce an A1 astrocyte phenotype, leading to neuronal and oligodendrocyte death, as well as synaptic collapse (Liddelow et al., 2017). The increased NF-κB expression in astrocytes also has the same effect (Xu et al., 2018).

Individual studies have demonstrated different points of view. For instance, the p50 subunit can regulate the balance of microglia M1/M2, and its dysfunction can lead to chronic inflammation (Taetzsch et al., 2015). Moreover, the expressions of TNF-α, IL-1β, and colony-stimulating factors can decrease the activation of microglia and astrocytes (Pérez-Otano et al., 1996). SE causes autophagic death of astrocytes through TNF-α, and the phosphorylation of p65/RelA-Ser529 (Ryu et al., 2011b) and IL-1 has an inhibitory effect on pentylenetetrazole (PTZ)-induced (Miller et al., 1991) or kindling-induced (Sayyah et al., 2005) seizures. Andrzejczak et al. suggested that the different functions of pro-inflammatory factors may depend on their concentration and the type of receptors involved in the response (Andrzejczak, 2011).

## Nuclear Factor Kappa B, Endothelial Cells, the Blood-Brain Barrier, and Drug-Resistant Epilepsy

Nuclear factor kappa B is expressed in neurons, glial cells, and endothelial cells. The expressions of cytokines (Liu X. X. et al., 2020), receptors (Kamali et al., 2020), enzymes, and P-gp (Ke et al., 2019) are altered in endothelial cells just like in neurons and glial cells, and similar effects are observed.

Under physiological conditions, activating NF-κB can protect the BBB (Ridder et al., 2015). Dysfunctions of NF-κB threonine 435 phosphorylation and endothelial NOS in endothelial cells (Kim et al., 2019) can lead to vasogenic edema with neutrophil infiltration and loss of astrocytes (Kim et al., 2012). After SE, resident microglia are the main source of chemokine (C-C motif) ligand 2. Due to the destruction of the BBB integrity, chemokine (C-C motif) ligand 2 and CC chemokine receptor 2 can recruit monocytes and macrophages, leading to the infiltration of white blood cells and the formation of vasogenic edema (Kim et al., 2020). Excitotoxicity is a central pathological pathway in epilepsy with BBB dysfunction (Barna et al., 2020). Seizures may modulate the BBB function, affect astrocytes and the innate immune system, and ultimately alter neuronal networks (Löscher and Friedman, 2020). This may, in turn, lead to changes in the central nervous system (Marchi et al., 2012).

Several studies have shown that BBB dysfunction is one of the main causes of drug resistance in epilepsy (Ogaki et al., 2020). P-gp is a member of the ATP-binding box superfamily of transmembrane proteins and is targeted by several pharmacological barriers (Ambudkar et al., 2006). Specifically, its expression increases through different signaling pathways by L-glutamate stimulation (Zhu and Liu, 2004). It binds to drugs and ATP, which, in turn, pumps the drugs out of the cell, reducing the intracellular concentration of drugs and making the cell resistant to them. Ke et al. (2019) found that P-gp was overexpressed in bEnd.3 cells and showed that endothelial cells play a role in the development of drug-resistant epilepsy. The p65 subunit can be combined with the Multi-Drug Resistance Gene 1 to promote the expression of P-gp on the cell membrane (Geick et al., 2001; Yu et al., 2011; Deng et al., 2019). The activation of NF-κB enhances the spillover transport of antiepileptic drugs across the BBB (Yu et al., 2011). The NMDA receptor and COX-2 can increase the expressions of P-gp in the BBB after SE (Bauer et al., 2008). P-gp is also expressed in glial cells (Wang X. et al., 2018) and neurons (Deng et al., 2019; Merelli et al., 2019) and may have an abnormal expression after SE. Interestingly, the overexpression of P-gp may be one of the mechanisms for the development of drug-resistant epilepsy that is closely associated with sudden unexpected death observed in epilepsy (Auzmendi et al., 2021). However, specific inhibitors of P-gp are known to produce unpredictable toxicity in clinical trials (Deng et al., 2009).

## Effects of Existing Treatments on Nuclear Factor Kappa B

In animal experiments, it has been found that directly inhibiting the activity of NF-κB will have a certain effect on epilepsy. Pyrrolidine dithiocarbamate salt (PDTC) is a drug that can specifically antagonize NF-κB (Soerensen et al., 2009). Yu et al. (2011) found that PDTC pretreatment prolonged the seizure onset time, decreased the P-gp overexpression, and failed to prevent brain cell loss in KA-induced rats. Shin et al. (2004) reported that PDTC prevented hippocampal neuronal loss in the KA-induced seizure model, and the use of a low dose of PDTC can almost completely protect from lesions in the piriform cortex (Soerensen et al., 2009) and attenuate the microglial activation (Lv et al., 2014). Treatment with 150 mg/kg PDTC before and after SE significantly increased the mortality rate to 100% (Soerensen et al., 2009). SN50 peptide can also inhibit the activity of NF-κB. It reduces the vasogenic edema caused by epilepsy (Kim and Kang, 2017) and inhibits the NF-κB-induced expression of P-gp in rat brain capillaries (Yu et al., 2011). The mRNA and protein levels of P-gp were remarkably reduced when NF-κB p65 was knocked down by siRNA transfections (Ke et al., 2019). Furthermore, NF-κB “decoy” inhibited COX-2 expression in an epileptic rat brain (Xu et al., 2020).

Some non-selective drugs can also act through the NF-κB pathway. Aspirin, a member of non-steroidal anti-inflammatory drugs, can reduce the content of NF-κB and protect against corticohippocampal neurodegeneration in an epilepsy model induced by PTZ (Abd-Elghafour et al., 2017). Dimethyl fumarate, an activator of Nrf2, downregulated the expression of inflammatory factors (NF-κB) with the reduction of the seizure score, percentage of kindled rats, and neurological damage score (Singh et al., 2019). Edaravone can protect hippocampal neurons from damage in KA-induced epilepsy rats by upregulating Nrf2 and downregulating NF-κB (Liu et al., 2018). Valproic acid can reduce the death of neural progenitor cells through the activation of NF-κB signaling (Go et al., 2011), which indicates that NF-κB can promote neuronal survival mentioned above. Long-term use of Antiseizure medications, such as phenytoin (Zhou et al., 2015), valproic acid (Rao et al., 2007), carbamazepine (Yu et al., 2017), pregabalin (Nader et al., 2018; Attia et al., 2019), and diazepam (Firdous et al., 2021), can reduce the amount of NF-κB in animal tissues or cells to protect neurons and suppress inflammation. Carbamazepine can also regulate the expression of P-gp, which may be related to drug-resistant epilepsy (Yu et al., 2017). From these, we found that different antiepileptic drugs may play different roles in the NF-κB pathway. This may be related to the fact that phosphorylation at different sites of different NF-κB molecules can cause different effects.

The discovery of new therapy is also important. Extracts from plants, such as curcumin (Karimian et al., 2017), rosaceae (Firdous et al., 2021), hyperforin (Lee et al., 2014), asiatic acid (Wang Z. H. et al., 2018), and gastrodin (Zhou et al., 2015), can downregulate neuroinflammation by preventing NF-κB phosphorylation in animal models, and their mechanisms of action are similar to that of valproate (Chen et al., 2018). Sitagliptin and nilotinib (Nader et al., 2018; Attia et al., 2019), neither of which is related to epilepsy treatment, can be used to treat epilepsy *via* the NF-κB pathway when used together with anticonvulsants. Anti-inflammatory miR-146a extended the latency of generalized convulsions, reduced the seizure severity, and decreased the expressions of its target mRNAs (IRAK-1 and TRAF-6) and NF-κB (Tao et al., 2017). The above treatment methods are not used in clinical practice, but the ketogenic diet, originally developed for the treatment of epilepsy in non-responder children, exhibits anti-inflammatory effects by inhibiting NF-κB (Pinto et al., 2018). There are no experiments on p65-related gene knockout mice, because mice lacking the p65 gene die within embryonic days 14–15 (Won et al., 1999).

## Conclusion

Nuclear factor kappa B is a transcription factor whose upregulation is responsible for increasing the expression of pro-inflammatory cytokines during neuronal excitability and hippocampal gliosis. It can be divided into two subfamilies and both include many factors, but the dimers that have been discovered to play a role in epilepsy do not contain all the molecules. Because the existing studies have shown that phosphorylation of different molecules, or phosphorylation at different sites of the same molecule, may exert different physiological functions, the identification of the effects of different molecules is particularly important. It is undeniable that in epilepsy, the upregulation of NF-κB can play a role in protecting neurons, and there is no doubt that the inflammatory response and antioxidant imbalance caused by NF-κB activation during epilepsy has a harmful effect on the central nervous system. Further research is needed to identify the role of different NF-κB molecules and different dimers in epilepsy, and to find new and more stable compounds that specifically inhibit one or more molecules of NF-κB to treat epilepsy. Hopefully 1 day, these drugs can be applied clinically and can bring good news to patients with epilepsy.

## Author Contributions

MC wrote the manuscript. WL provided the ideas and finally revised the manuscript. Both authors contributed to the article and approved the submitted version.

## Conflict of Interest

The authors declare that the research was conducted in the absence of any commercial or financial relationships that could be construed as a potential conflict of interest.

## Publisher’s Note

All claims expressed in this article are solely those of the authors and do not necessarily represent those of their affiliated organizations, or those of the publisher, the editors and the reviewers. Any product that may be evaluated in this article, or claim that may be made by its manufacturer, is not guaranteed or endorsed by the publisher.
